# Reactive Carbonyls Induce TOR- and Carbohydrate-Dependent Hormetic Response in Yeast

**DOI:** 10.1155/2020/4275194

**Published:** 2020-03-12

**Authors:** Halyna Semchyshyn

**Affiliations:** Department of Biochemistry and Biotechnology, Vasyl Stefanyk Precarpathian National University, 57 Shevchenko Str., 76018 Ivano-Frankivsk, Ukraine

## Abstract

The induction of the beneficial and detrimental effects by reactive carbonyl species in yeast has been investigated. In this study, we have presented evidence that glyoxal and methylglyoxal at low concentrations were able to induce a hormetic adaptive response in glucose-grown but not fructose-grown yeast. The hormetic effect was also TOR-dependent. The mutation in genes encoding either TOR1 or TOR2 protein makes yeast highly sensitive to both *α*-dicarbonyls studied. Simultaneous disruption of TOR1 and TOR2 resulted in higher yeast sensitivity to the *α*-dicarbonyls as compared to parental cells, but double mutant survived better under carbonyl stress than its single mutant counterparts. The data obtained are consistent with the previous works which reported high toxicity of the *α*-dicarbonyls and extend them with the report on the beneficial TOR-dependent hormetic effect of glyoxal and methylglyoxal.

## 1. Introduction

A large family of highly reactive organic molecules of short or intermediate length (3–9 carbons) containing at least one carbonyl group are referred to as reactive carbonyl species (RCS). Reactive carbonyls can be of both exogenous and endogenous origin. Some products of organic-pharmaceutical chemistry, industrial pollutants, cigarette smoke, food additives, and components of browned food are widespread exogenous RCS that can easily enter a cell [1–3]. Endogenously, the generation of RCS is associated with normal metabolic processes, particularly enzymatic and nonenzymatic reactions of carbohydrates and lipids [4, 5]. Both exogenous and endogenous reactive carbonyls demonstrate a large variety of effects and may have a dual biological impact; however, mainly they are known for their harmful influence [6]. Either derived from the environment or endogenously produced reactive carbonyls appear to be toxic because of their ability to modify structure/function of biomolecules and promote the formation of poorly degraded adducts or crosslinks collectively referred to as advanced glycation end products. In most cases studied, the level of RCS and glycation products substantially increases with aging, may disturb cellular metabolism, and accelerate pathological processes [3, 7].

On the other hand, the beneficial role of RCS is also known. Some of the reactive carbonyls are implicated in immune response, cellular signaling, and regulation of gene expression. Showing anticancer, antimicrobial, and antiviral activities, certain RCS are suggested to be promising as potential therapeutic agents [8, 9]. Increasing evidence indicates that different reactive species (e.g., oxygen-, nitrogen-, and sulfur-containing molecules) trigger hormetic response [10, 11]; however, possible involvement of RCS has not been intensively investigated. It is clear from many studies that RCS can modulate cell signaling pathways, including the stress responses, proapoptotic processes, enzyme activities, and transcription factor functions in different experimental models [9, 12–15].

The target of rapamycin (TOR) is a highly conserved signaling pathway that is involved in a cell response to various extracellular and intracellular stimuli [16, 17]. Obviously, advanced glycation end products modulate/regulate many metabolic, physiological, and pathological events through the TOR network [7]. However, the interplay between TOR and RCS has attracted only minor attention. In our previous experiments, yeast parental strain and its derivatives defective in the TOR proteins demonstrated different intracellular levels of RCS [18] that were consistent with the previous suggestion that TOR inhibition suppressed the generation of RCS [19]. Here, we used TOR-deficient strains to investigate whether reactive carbonyls would lead to a hormetic adaptive response in yeast.

## 2. Materials and Methods

### 2.1. Yeast Strains and Chemicals

The *Saccharomyces cerevisiae* strains were used as follows: JK9-3da (wild-type *MAT ***a ***leu2–3*, *112 ura3–52 rme1 trp1 his4 HML ***a**) [20] and its derivatives MH349-3d (JK9-3da, *tor1::LEU2-4*) [21], SH121 (JK9-3da, *ade2 tor2::ADE2-3/YCplac111::tor2-21*^*ts*^) [22], and SH221 (JK9-3da, *ade2 his3 HIS4 tor1::HIS3 tor2::ADE2-3/YCplac111::tor2-21*^*ts*^) [23], kindly provided by Professor Michael Hall (University of Basel, Switzerland). Chemicals were obtained from Sigma-Aldrich Chemical Co. (USA) and Fluka (Germany).

### 2.2. Growth Conditions and Stress Induction

Yeast cells were grown at 28°С with shaking at 175 r.p.m. in a liquid medium containing 1% yeast extract, 2% peptone, and 1% sucrose. For the experiments, the cultures after 24 h growth were split into two portions and diluted to about 75 × 10^6^ cells/mL in a medium containing 1% yeast extract, 2% peptone, and 2% glucose (YPD). Glucose was substituted for fructose in the respective experiments. In all diluted cultures, cells were grown under the conditions mentioned above for an additional 24-h period.

**Figure 1:**
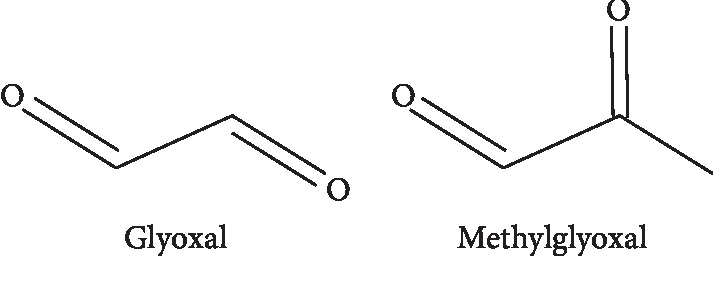
Chemical structures of *α*-dicarbonyl compounds: glyoxal and methylglyoxal.

Aliquots of the main cultures (glucose- and fructose-grown) were exposed to different concentrations of glyoxal or methylglyoxal (Figure 1) followed by their incubation at 28°C for 1 h. Control cells were incubated under the same conditions but without stressing agents. After the incubation, cells from experimental or control cultures were collected by centrifugation (5 min, 8000 g) and washed with 50 mM of potassium phosphate buffer (pH 7.0).

### 2.3. Reproductive Ability

Yeast reproductive ability was analyzed by plating in triplicate on YPD agar after proper dilution. The plates were incubated at 28°C for 3 days and the colony-forming units (CFU) were counted [24]. Reproductive ability was expressed as a percentage of the total number of cells plating on YPD agar. Experimental data are expressed as the mean value of 3–6 independent experiments ± the standard error of the mean (SEM).

## 3. Results and Discussion

Mild stress and hormesis are the most likely explanations for the beneficial effects of low doses of toxic substances [25–27]. The hormetic response is determined by the substance nature, physiological state of the organism, and specificity of downstream targets influenced [28–31]. A wide variety of stressing agents have beneficial hormetic effects, however, the potential role of reactive carbonyls has not been intensively investigated. To study whether RCS would lead to a hormetic adaptive response in yeast and TOR pathway would be involved in RCS-induced carbonyl stress, we used *S. cerevisiae* JK9-3da (*parent strain*) and its TOR mutants: TOR1 and TOR2 single mutants (Δ*tor1* and Δ*tor2*) as well as TOR1 TOR2 double mutant (Δ*tor1*Δ*tor2*). The yeast was grown on glucose or fructose since we have recently found that carbon substrate in cultivation medium was an important factor of the endogenous generation of RCS and determined yeast hormetic phenotype [18, 30, 32]. Monosaccharides are usually used as carbon and energy source for yeast growth. Normal metabolic processes like glycolysis/fermentation are tightly associated with the generation of *α*-dicarbonyl compounds [5]. Reactive carbonyls, and *α*-dicarbonyl compounds, in particular, are found to be about 20,000-fold more reactive than reducing carbohydrates [4]; therefore *α*-dicarbonyls like glyoxal and methylglyoxal (Figure 1) can be highly toxic compounds [33, 34].

**Figure 2:**
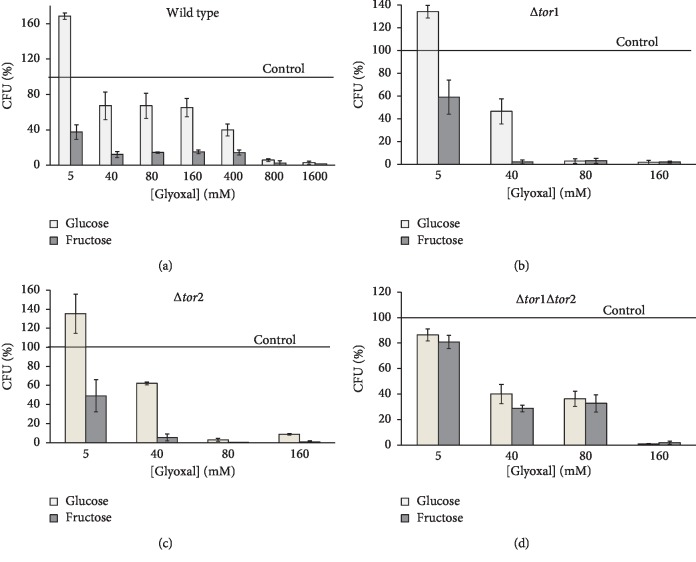
Effect of glyoxal on the reproductive ability of *S. cerevisiae* JK9-3da wild type (a) and its mutants *TOR1* (b), *TOR 2* (c), and *TOR1 TOR2* (d). Results are shown as the mean ± SEM (*n* = 3–6).

The impact of different concentrations of glyoxal on yeast reproductive ability is shown in Figure 2. The biphasic dependence, characterized by low-dose stimulation and high-dose repression of yeast colony growth, has been found for glucose-grown cultures (Figures 2(a)–2(c)) with the exception of the TOR1 TOR2 double mutant (Figure 2(d)). Wild-type cells and both single mutants grown in glucose-containing medium demonstrated the peak hormetic response at 5 mmol/L glyoxal. A hormetic effect usually can be observed under mild stress conditions as an increase in biological function between the ranges of 30–60% [35]. Our data are in good agreement with the above-mentioned: at the hormetic concentration of glyoxal, wild type and both single knockouts showed about 168%, 134%, and 135% of the initial reproductive ability (without glyoxal), respectively. The parameter decreased with further increasing glyoxal concentration. At 40 mmol/L glyoxal, yeast colony growth dropped to about 46–67% of the control reproductive ability. At the highest glyoxal concentrations used (≥80 mmol/L), the reproductive ability of the TOR1 and TOR2 single mutants dramatically decreased to the lowest values; and very few of the knockout cells were able to survive after the treatment. Parental strain demonstrated similar to the single mutants sharp reduction of reproductive ability only after cell exposure to 0.8–1.6 mol/L glyoxal.

Fructose-grown, unlike glucose-grown cells, did not show a hormetic response at any glyoxal concentration used. Moreover, the reproductive ability of fructose-grown wild type and two single mutants under glyoxal-induced stress was by several-fold lower than that of respective glucose-grown yeast (Figures 2(a)–2(c)). The only exception occurs for the two highest glyoxal concentrations used at which very few of both the studied cell groups (glucose- and fructose-grown) survived.

In the previous reports, the defensive effect of fructose against stressful challenges has been described [36–39]. We have suggested that fructose via generation of reactive species was capable of provoking a mild/temporary stress that resulted in the acquisition of resistance to severe stress [40, 41]. The suggestion was prompted by the fact that fructose-grown compared to glucose-grown yeast demonstrated higher survival after exposure to low concentrations of hydrogen peroxide [42]. Our previous data also demonstrated that in fructose-grown cells, the peak hormetic response was shifted to higher concentrations of H_2_O_2_ as compared to glucose-grown yeast [30, 32].

Unlike the abovementioned studies, the data presented here demonstrated no beneficial impact of fructose on yeast survival under glyoxal-induced carbonyl stress (Figures 2(a)–2(c)). Moreover, glyoxal was more toxic for fructose-grown parental cells and single mutants than respective glucose-grown yeast. Surprisingly, this was not the case for the TOR1 TOR2 double mutant; both types of Δ*tor1*Δ*tor2* cells studied (glucose- and fructose-grown) demonstrated virtually the same survival after exposure to glyoxal (Figure 2(d)). Interestingly, at certain glyoxal concentrations, the double knockout had reproductive ability several orders of magnitude higher than its single mutant counterparts. It was even more surprising that fructose-grown double mutant after exposure to 5–80 mmol/L glyoxal showed about 2-fold higher colony growth than fructose-grown wild type (Figures 2(a) and 2(d)).

**Figure 3:**
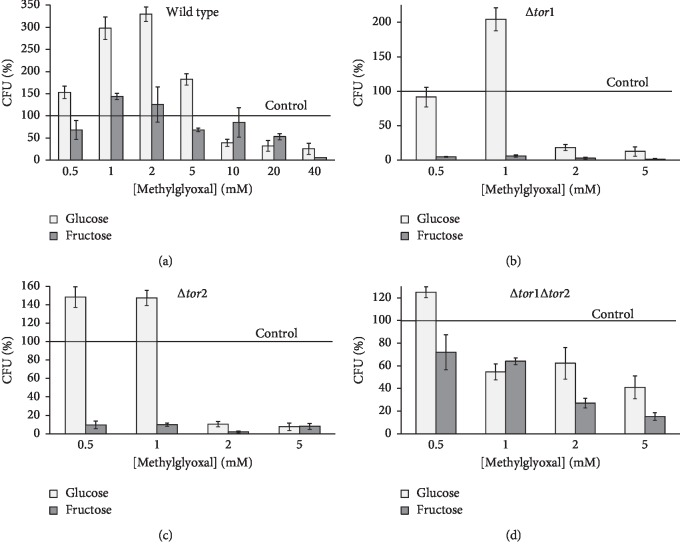
Effect of methylglyoxal on the reproductive ability of *S. cerevisiae* JK9-3da wild type (a) and its mutants *TOR1* (b), *TOR2* (c), and *TOR1 TOR2* (d). Results are shown as the mean ± SEM (*n* = 3–6).

Exposure to methylglyoxal resulted in similar patterns of carbohydrate- and TOR-dependent colony growth of the yeast strains (Figure 3). Glucose-grown wild type (Figure 3(a)) demonstrated the peak of hormetic response at 0.5–5 mmol/L methylglyoxal with reproductive ability about 1.5-3-fold higher than that in control cells (without methylglyoxal). In comparison, fructose-grown parental cells showed a weak tendency to increase their reproductive ability at low concentrations of methylglyoxal (Figure 3(a)). Both the studied glucose-grown TOR1 and TOR2 single mutants demonstrated the peak hormetic response (148–200%) after the yeast treatment with 0.5–1 mmol/L methylglyoxal, while it was not the case for their fructose-grown counterparts at any methylglyoxal concentration used (Figures 3(b) and 3(c)). In general, the TOR1 and TOR2 single knockouts grown in glucose-containing medium significantly lost their viability after exposure to 2–5 mmol/L methylglyoxal. Fructose-grown single mutants demonstrated extremely low survival after their incubation with methylglyoxal at any concentrations used. High toxicity of exogenous glyoxal and methylglyoxal towards single knockouts (Figures 2(b), 2(c), 3(b), and 3(c)) correlates with their relatively low total intracellular level of *α*-dicarbonyl compounds [18] and a remarkably high activity of glyoxalase I detoxifying *α*-dicarbonyls [32]. The TOR1 and TOR2 proteins share common functions and therefore can compensate for the loss of each other [43, 44]. This could explain a similar behavior of the single mutants under the same experimental conditions (Figures 2(b), 2(c), 3(b), and 3(c)). The double mutant treated with either glyoxal (Figure 2(d)) or methylglyoxal (Figure 3(d)) exhibited a phenotype not evident in either single mutant (Figures 2(b), 2(c), 3(b), and 3(c)). There may be some compensatory mechanisms for the simultaneous lack of TOR1 and TOR2 since generally, the reproductive ability of the double knockout under stressful conditions was significantly higher than that of its single mutant counterparts.

## 4. Conclusion

Glyoxal and methylglyoxal induce TOR- and monosaccharide-dependent hormetic adaptive response in *S. cerevisiae*. The mutations in either TOR1 or TOR2 gene make yeast highly sensitive to both *α*-dicarbonyls. Nevertheless, methylglyoxal is more toxic towards yeast cells than glyoxal, and hormetic concentrations of methylglyoxal are lower than those of glyoxal. These data correspond well to the observations by Hoon and colleagues [45]. The TOR1 and TOR2 single mutants indicate a similar behavior under the same experimental conditions, which can be explained by the well-documented fact that TOR1 and TOR2 share common functions in the yeast. Simultaneous mutations in the TOR1 and TOR2 genes make yeast more susceptible to RCS than parental cells, but double mutant survives better under RCS-induced carbonyl stress than its single mutant counterparts. These results suggest the presence of compensatory mechanism(s) when both TOR1 and TOR2 are depleted. The information obtained could be useful to investigate RCS-mediated nonenzymatic processes and their involvement in metabolic disorders and related pathological processes.

## Data Availability

The data that support the findings of this study are (1) originally obtained in this study and (2) literature data available in respective sources referred to in the manuscript.
